# Genome-Wide Analysis of MDHAR Gene Family in Four Cotton Species Provides Insights into Fiber Development via Regulating AsA Redox Homeostasis

**DOI:** 10.3390/plants10020227

**Published:** 2021-01-25

**Authors:** Fangfang Zhou, Bowen Zheng, Fei Wang, Aiping Cao, Shuangquan Xie, Xifeng Chen, Joel A. Schick, Xiang Jin, Hongbin Li

**Affiliations:** 1 Key Laboratory of Xinjiang Phytomedicine Resource and Utilization of Ministry of Education, College of Life Sciences, Shihezi University, Shihezi 832003, China; zhouff@stu.shzu.edu.cn (F.Z.); zhengbw@stu.shzu.edu.cn (B.Z.); feiw@shzu.edu.cn (F.W.); caoaiping@shzu.edu.cn (A.C.); xiesq@shzu.edu.cn (S.X.); cxf_cc@shzu.edu.cn (X.C.); 2 Ministry of Education Key Laboratory for Ecology of Tropical Islands, College of Life Sciences, Hainan Normal University, Haikou 571158, China; 3 Genetics and Cellular Engineering Group, Institute of Molecular Toxicology and Pharmacology, Helmholtz Zentrum Muenchen, 85764 Neuherberg, Germany; joel.schick@helmholtz-muenchen.de

**Keywords:** *Gossypium*, MDHAR gene family, fiber growth, ascorbate recycling, H_2_O_2_, redox regulation

## Abstract

Monodehydroasorbate reductase (MDHAR) (EC1.6.5.4), a key enzyme in ascorbate-glutathione recycling, plays important roles in cell growth, plant development and physiological response to environmental stress via control of ascorbic acid (AsA)-mediated reduction/oxidation (redox) regulation. Until now, information regarding *MDHAR* function and regulatory mechanism in *Gossypium* have been limited. Herein, a genome-wide identification and comprehensive bioinformatic analysis of 36 *MDHAR* family genes in four *Gossypium* species, *Gossypium arboreum*, *G. raimondii, G. hirsutum*, and *G. barbadense*, were performed, indicating their close evolutionary relationship. Expression analysis of *GhMDHARs* in different cotton tissues and under abiotic stress and phytohormone treatment revealed diverse expression features. Fiber-specific expression analysis showed that *GhMDHAR1A/D*, *3A/D* and *4A/D* were preferentially expressed in fiber fast elongating stages to reach peak values in 15-DPA fibers, with corresponding coincident observances of MDHAR enzyme activity, AsA content and ascorbic acid/dehydroascorbic acid (AsA/DHA) ratio. Meanwhile, there was a close positive correlation between the increase of AsA content and AsA/DHA ratio catalyzed by MDHAR and fiber elongation development in different fiber-length cotton cultivars, suggesting the potential important function of MDHAR for fiber growth. Following H_2_O_2_ stimulation, *GhMDHAR* demonstrated immediate responses at the levels of mRNA, enzyme, the product of AsA and corresponding AsA/DHA value, and antioxidative activity. These results for the first time provide a comprehensive systemic analysis of the *MDHAR* gene family in plants and the four cotton species and demonstrate the contribution of MDHAR to fiber elongation development by controlling AsA-recycling-mediated cellular redox homeostasis.

## 1. Introduction

Reactive oxygen species (ROS) are important signaling molecules that play significant roles in plant development, cell growth and in response to environmental stresses and phytohormone signaling [1,2,3,4,5]. To counteract excessive ROS and the detrimental effects of cellular oxidation, plants have evolved corresponding defense mechanism for detoxification of ROS by controlling cellular reduction/oxidation (redox) balance. In this, the ascorbate-glutathione (Asc-GSH) cycle, also namely Asc recycling pathway, is regarded as the critical pathway that mediates the antioxidative molecule of Asc as a coenzyme to eliminate redundant ROS by involving them in the enzymatic reactions. It also stabilizes cellular redox status to play a multifunction role in controlling cell growth and plant development [6,7]. Of the Asc antioxidant system enzymes, ascorbate peroxidase (APX, EC1.11.1.11) is the prototypical ROS-scavenging enzyme utilizing ascorbic acid (AsA) as substrate to reduce hydrogen peroxide (H_2_O_2_) to water [4,8]. As a byproduct of the reaction, the generated monodehydroascorbate acid (MDHA, an oxidative form of Asc) is then reduced to AsA by monodehydroascorbate reductase (MDHAR, EC1.6.5.4) to balance the AsA pool in cells by GSH-coupled continuous enzymatic reactions [6,9].

As evinced by genetic experiments, Asc is an important abundant antioxidant that executes a vital role in detoxifying cellular excessive ROS by enzymatic reactions [6,9], regulating plant growth and development [10,11,12] as well as controlling cellular processes including cell growth and cell division [13,14,15,16]. For Asc biosynthesis, there are four proposed pathways, including the Smirnoff–Wheeler (SW) pathway, the *myo*-inositol (MI)-dependent d-glucuronate pathway, the d-galacturonic acid pathway and the recycling pathway by the Foyer–Halliwell–Asada cycle [9,17,18,19]. Among them, the recycling pathway catalyzed by MDHAR is critical for control of the cellular redox status by regeneration of AsA [20,21]. Attempts to increase Asc levels via MDHAR have been widely reported. The AsA content and the AsA/DHA ratio are closely linked with MDHAR activity in tomato [22]. Overexpression of the *LeMDHAR* gene significantly elevated AsA level and decreased the H_2_O_2_ content in tomato (*Lycopersicon esculentum* Mill.) [23]. *Chlamydomonas reinhardtii CrMDHAR1* is a ROS-inducing gene and its overexpression elevated the AsA level and AsA/DHA ratio to resist photooxidative stress [24]. Transgenic tobacco plant lines overexpressing Arabidopsis *AtMDHAR1* correlated enrichment of MDHAR enzyme activity and accumulation of AsA content with significantly enhanced tolerance against environmental stress [25].

As a thiol-group-containing flavin adenine dinucleotide (FAD) monomeric enzyme of the AsA-GSH cycle, MDHAR belongs to a multigene family and has been reported to locate in different organelles such as the chloroplast [23,26], mitochondria and peroxisome [27,28,29], and in cytosol [30], as well as both mitochondria and chloroplasts [31]. The *MDHAR* cDNA has been cloned from diverse plant species containing pea (*Pisum sativum* L.) [32], tomato (*Lycopersicon esculentum* Mill.) [33] and spinach (*Spinacea oleracea*) [34]. The MDHAR proteins were also purified from cytosol of cucumber (*Cucumis sativus*), soybean (*Glycine max*) and potato (*Solanum tuberosum*) [35,36,37], the mitochondria of potato [38], and chloroplasts of spinach (*Spinacia oleracea*) [34]. Three *Physcomitrella patens* PpMDHAR cytosolic isoforms indicated different response patterns with increased accumulation of *PpMDHAR1* and *PpMDHAR3* to salt and osmotic stresses, and normal level of *PpMDHAR2* to chilling and oxidative stresses, showing that cytosolic MDHARs are essential for land plants to adapt to dry terrestrial conditions [39]. In Arabidopsis, there are six MDHAR polypeptides, namely AtMDHAR1 to AtMDHAR6, with the different organelle distributions of AtMDHAR1 and AtMDHAR4 in peroxisomes, AtMDHAR2 and AtMDHAR3 in cytosol, AtMDHAR5 in mitochondria and AtMDHAR6 in chloroplasts [40].

Numbers of reports have revealed the important role of MDHAR in resisting abiotic stress in different plants such as arabidopsis [41], wheat [42], tomato [28], etc., by controlling the cellular redox homeostasis as a negative regulator to reduce the cellular ROS level. The multifunctionality of MDHAR has also been studied, including detoxification of 2,4,6-trinitrotoluene (TNT) toxicity [43], reduction of phenoxyl radicals [44], increase of *Saccharomyces cerevisiae* fermentative capacity [45], strengthening of disease resistance [42], as well as plant growth and development of yield maintenance [46], postgerminative growth [29] and fruit ripening [47].

Cotton cultivars are important crop plants worldwide for their supply of fibers used as raw materials to the textile industry. Fiber growth and development is closely correlated with the final fiber quality [4,48]. A genome-wide analysis of the genes controlling fiber development is an effective method for elucidation of the molecular mechanism, and thus for the potential improvement, of fiber quality [49]. With regard to the Asc metabolic pathway in cotton fiber growth, our previous investigations have shown that, ascorbate peroxidase (APX) appeared with an ethylene-induced expression and participated in fiber cell fast elongation development by modulating ROS homeostasis [4,48]. Moreover, the Asc de novo biosynthesis gene *GhVTC1* is the key factor to promote cell elongation under control of ethylene [50], while the *myo*-inositol-1-phosphate synthase (MIPS) located in the MI-dependent d-glucuronate pathway participated in cell elongation by providing the precursor MI to contribute to Asc biosynthesis [51]. Ascorbate oxidase (AO) is a positive regulator to enhance tobacco cell elongation through generation of H_2_O_2_-mediated apoplast oxidation [16]. Together, these results suggest an intimate connection between Asc metabolic genes and cellular ROS balance in fibers. Currently, information regarding *MDHAR* genes and their functions, as well as the regulatory mechanism involved in fiber development in *Gossypium*, are largely unknown. In the present study, by a genome-wide identification in four cotton species of *G. arboreum*, *G. raimondii*, *G. hirsutum* and *G. barbadense*, a total of 36 *MDHAR* genes were obtained. A comprehensive systematic bioinformatic analysis, including chromosomal location, gene structure, motif distribution and evolutionary relationships, was performed indicating rapid evolution and close evolutionary relationships. *G. hirsutum GhMDHARs* that showed diverse expression patterns under abiotic stress, and phytohormone treatment demonstrated a close correlation with fiber growth by increasing MDHAR activity, AsA content and AsA/DHA ratio, possibly via involvement of AsA-mediated H_2_O_2_ signaling pathway.

## 2. Results

### 2.1. Identification and Chromosome Location of MDHAR Genes of Four Gossypium Species

By searching the genome of *G. arboreum*, *G. raimondii*, *G. hirsutum* and *G. barbadense* using arabidopsis MDHAR proteins as queries with the BlastP procedure, a total of 36 *MDHAR* genes were identified, with six in diploid cotton (*G. arboreum* and *G. raimondii*), and twelve in allotetraploid cotton (*G. barbadense* and *G. hirsutum*) (Supplementary Table S1). The allotetraploid cotton species have twice the *MDHAR* members in comparison to the diploid cotton species. The *MDHARs* possess open reading frame (ORF) lengths ranging from 1305 to 1677 base pair (bp), the encoded proteins have 434 to 558 amino acids with theoretical molecular weight (MW) from 46.83 to 62.25 kDa and theoretical *p*I from 5.9 to 9.06, respectively (Supplementary Table S1).

**Figure 1:**
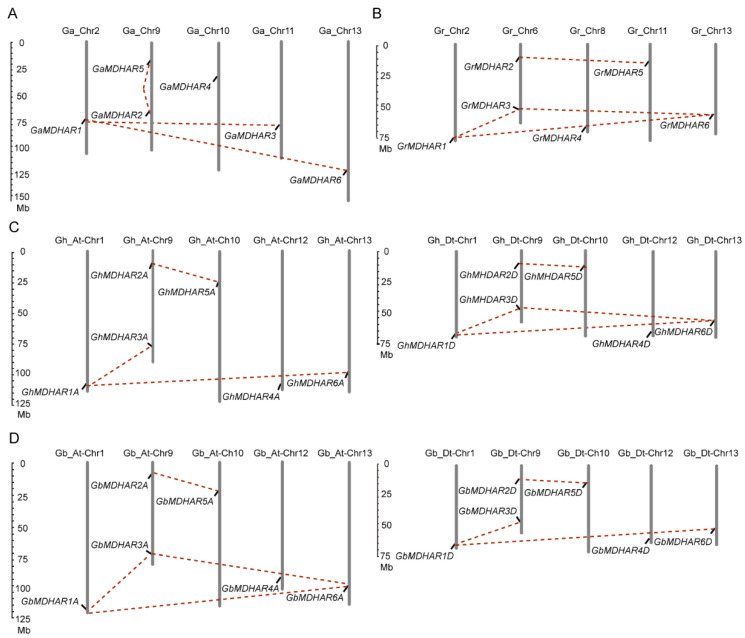
Chromosome distribution of *MDHAR* family genes in four *Gossypium* species. The chromosomal mapping of the *MDHAR* genes of A and D genome in diploid cotton of *G. arboreum* (**A**) and *G. raimondii* (**B**), and of A- and D subgenome in allotetraploid cotton of *G. hirsutum* (**C**) and *G. barbadense* (**D**), were illustrated. The red dashed lines indicate the segmentally duplicated gene pairs.

The *MDHARs* were distributed across different chromosomes, with locations on fivechromosomes of *G. arboreum* (AA genome, Chr 2, 9, 10, 11, and 13) and of *G. raimondii* (DD genome, Chr 2, 6, 8, 11, and 13). The distributions in allotetraploid cotton species showed similar patterns of orthologous *MDHARs* on the same chromosomes of *G. barbadense* and *G. hirsutum*, with locations on five At and five Dt subgenomes (Chr 1, 9, 10, 12, and 13) (Figure 1). The genomic locations also provide a clue to analyze gene duplication. In the At subgenome, four putative paralogous gene pairs, *MDHAR1*-*MDHAR3*, *MDHAR2*-*MDHAR5*, *MDHAR1*-*MDHAR6* and *MDHAR3*-*MDHAR6* (only in *G. barbadense* At subgenome), were found to be segmentally duplicated. The above four paralogous gene pairs were also identified as duplications in the Dt subgenome, with the absence of *MDHAR3*-*MDHAR6* in the *G. barbadense* Dt subgenome (Figure 1).

### 2.2. Phylogenetic and Evolutionary Analyses of MDHAR Members

**Figure 2:**
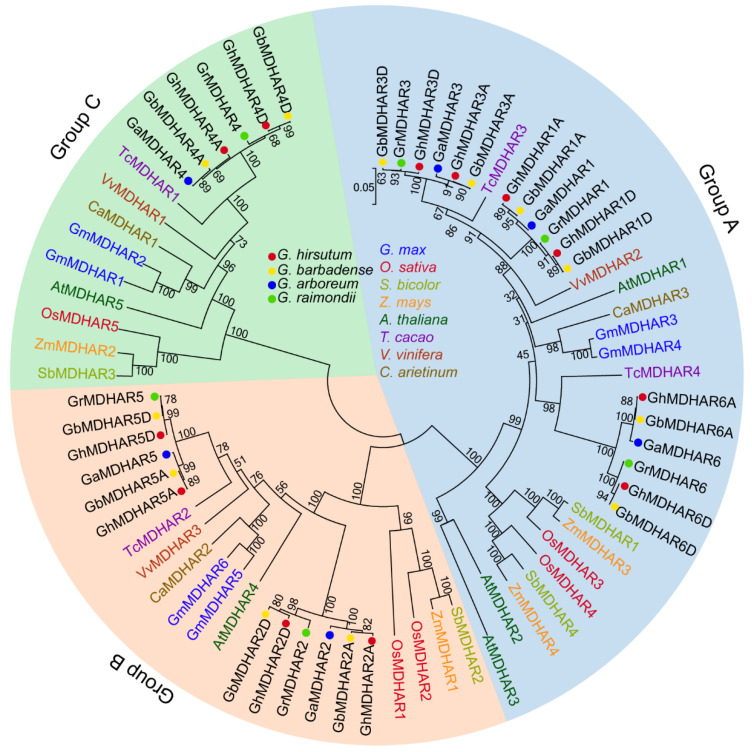
Phylogenetic analysis of *MDHAR* family genes in cotton and other plant species. A total of 70 MDHAR protein sequences from the four cotton species of *G. arboreum* (GaMDHARs), *G. raimondii* (GrMDHARs), *G. hirsutum* (GhMDHARs), and *G. barbadense* (GbMDHARs), and from another eight plant species including *Glycine max* (GmMDHARs), *Oryza sativa* (OsMDHARs), *Sorghum bicolor* (SbMDHARs), *Zea mays* (ZmMDHARs), *Arabidopsis thaliana* (AtMDHARs), *Theobroma cacao* (TcMDHARs), *Vitis vinifera* (VvMDHARs) and *Cicer arietinum* (CaMDHARs) were utilized for construction of a neighbor-joining (N—J) tree with 1000 bootstrap replicates, to generate three typical groups (Group A–C) that are indicated by different background colors. The different colored texts represent the distinct plant species, and the cotton MDHARs are marked with the diverse colored dots.

The *MDHAR* sequences from four cotton species of *G. arboreum*, *G. raimondii*, *G. barbadense* and *G. hirsutum*, and from another eight plants including *Glycine max*, *Oryza sativa*, *Sorghum bicolor*, *Zea mays*, *Arabidopsis thaliana*, *Theobroma cacao*, *Vitis vinifera* and *Cicer arietinum*, were used for construction of a neighbor-joining (N—J) tree, resulting in three typical groups (Group A–C). The orthologous *MDHARs* in cotton and cacao indicated a close relationship, which might be the reason that they are in the same family of *Malvaceae*. Cotton MDHAR homologs were classified into the same branches, with MDHAR1, 3, and 6 in group A, MDHAR 2 and 5 in group B, and MDHAR 4 in group C (Figure 2), demonstrating again the close relationship of gene duplication of *MDHARs* in the same groups.

The evolutionary relationship could be assessed by genetic synteny across related plant species. The syntenic analysis of *MDHAR* genes of the four cotton species was executed and visualized using Circos software (Supplementary Figure S1). Two paralogs in *G. arboreum*, one paralog in *G. raimondii,* nine paralogs in *G. barbadense* and nine paralogs in *G. hirsutum* had syntenic relationships. Meanwhile, the number of 47, 27 and 15 had syntenic relationships in Group A, Group B and Group C, respectively (Figure 2), showing that Group A had many syntenic genes compared with the other two groups, indicating the close evolutionary relationships of the *MDHAR* genes among the four cotton species. The evolutionary rate of *MDHAR* duplicated gene pairs was evaluated by the Tajima relative rate test, which indicated that *MDHAR1/6* and *MDHAR2/5* gene pairs showed accelerated evolutionary rate in the four *Gossypium* species, and the *MDHAR3/6* gene indicated accelerated evolutionary rate only in *G. hirsutum* (Supplementary Table S2), implying the potential functional divergence of these *MDHAR* gene pairs. Evolutionary selection analysis by nonsynonymous substitution rate/synonymous substitution rate (*Ka*/*Ks*) indicated that the ratio of all duplicated *MDHAR* gene pairs showed a value of <1, indicating all these *MDHAR* gene pairs were purifying selection and suggesting their rapid evolution (Supplementary Table S3).

### 2.3. Analyses of Gene Structure, Conserved Motif, and Promoter Cis-Acting Element of MDHAR Genes

**Figure 3:**
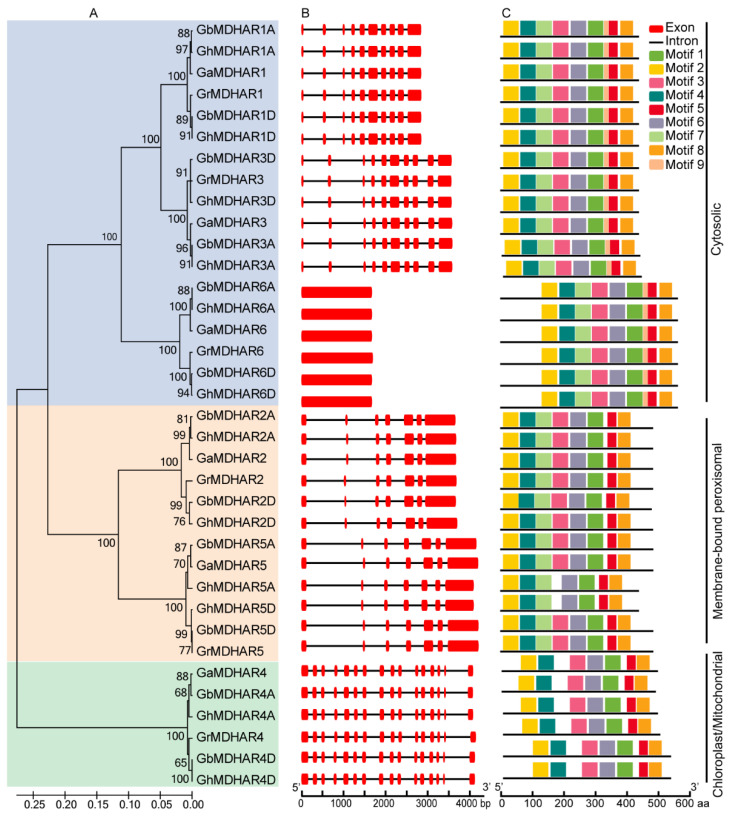
Gene organization and conserved motif analysis of the *MDHAR* gene family in four *Gossypium* species. (**A**) The cotton MDHARs were classified into three typical clusters which indicated the consistency with that in Figure 2. (**B**) Gene structures of the *MDHARs* are indicated with introns by black lines and the exons by red boxes. (**C**) A total of nine conserved motifs were discovered and indicated by different colored boxes with the specific distributions presented and the amino acid length displayed at the bottom. Both the intron/exon organizations and conserved motifs of the MDHARs demonstrated similar distributions in the same clusters.

To obtain more insights into the evolutionary relationship and potential functional diversity of *MDHAR* genes, exon/intron organization and conserved motif analyses were performed by Gene Structure Display Server (GSDS) and Multiple Em for Motif Elicitation) (MEME) programs respectively. The *MDHAR* genes could be divided into different branches (Figure 3A). The same homologous *MDHAR* genes in different cotton species were classified into the same branch and indicated a highly similar exon/intron arrangement, with the exception of *MDHAR6* that contained only one individual exon without intron (Figure 3B), suggesting conservation of the evolutionary relationship. All MDHARs presented high sequence similarity and contained a typical flavin adenine dinucleotide/nicotinamide adenine dinucleotide (FAD/NAD)-binding domain (Supplementary Figure S2), implying a conserved function in catalysis. As a result of nine discovered conserved motifs, nine, eight and seven conserved motifs were presented in MDHAR1, 3, and 6, MDHAR2 and 5, and MDHAR4, respectively, showing the corresponding predicted subcellular locations in the cytosol, membrane-bound peroxisomes, and chloroplast/mitochondria (Figure 3C). The detailed amino acid composition of the conserved motifs is presented in Supplementary Figure S3, suggesting the functional diversity of the different MDHARs. *Cis*-acting element analysis showed that the distributions in different *MDHAR* promoter regions are largely diverse, with the exception of the highly consistent composition of *MDHAR* promoters in A-subgenome but not in D-subgenome in the two allotetraploid species *G. hirsutum* and *G. barbadense*. Besides the core elements, regulatory elements related to development and responsiveness to stress and phytohormone were also discovered (Supplementary Figure S4) with detailed information of these elements in Supplementary Table S4, suggesting their potential function for plant growth and regulation by these factors.

### 2.4. Tissue-Specific and Fiber-Developmental Expression Analysis of G. hirsutum GhMDHARs

**Figure 4:**
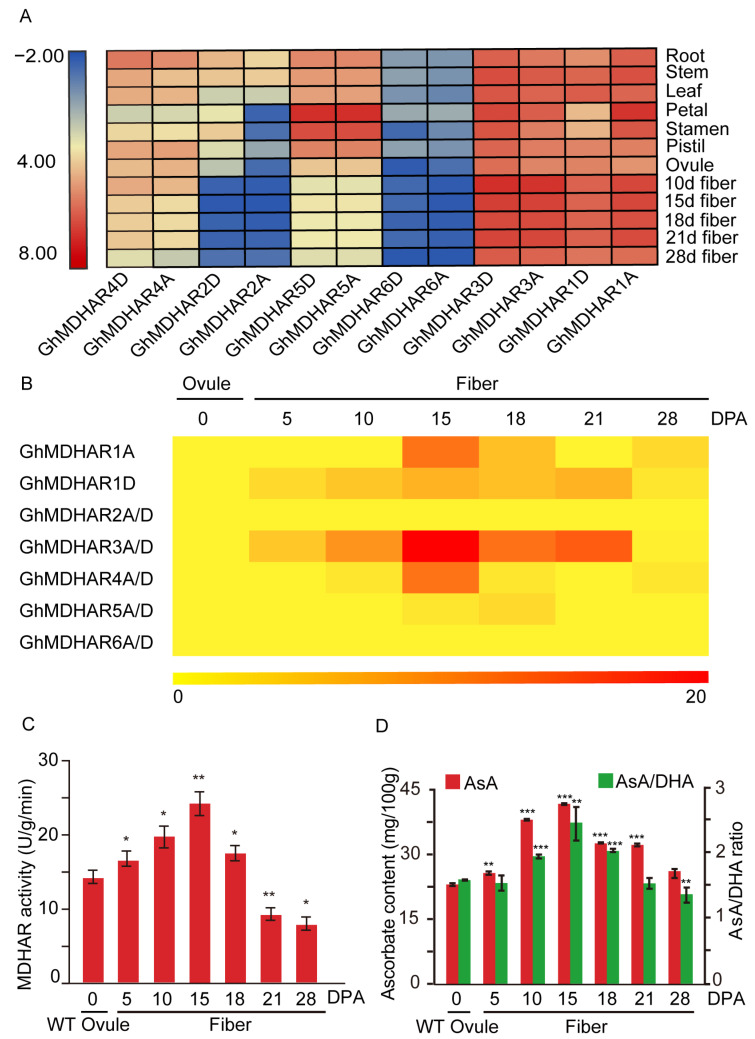
Tissue-specific and fiber-developmental expression analyses of *G. hirsutum GhMDHARs*. (**A**) Heatmap of expression levels of *GhMDHAR* genes in different tissues. The fragments per kilobase of exon per million reads mapped (FPKM) values in different tissues including root, stem, leaf, petal, stamen, pistil, ovule and fiber were obtained from the public transcriptomic data released at the website of NCBI and used to generate the heatmap. The colors of blue, yellow, and red indicate the low, moderate and high expression levels respectively. (**B**) qRT-PCR-based heatmap of expression levels of *GhMDHAR* genes during fiber developmental stages. The 0-DPA ovules and fibers at different stages of 5, 10, 15, 18, 21 and 28 DPA, were collected and used for RNA extract to perform the qRT-PCR assay to analyze the expression levels of *GhMDHAR* genes. *GhUBQ7* gene was utilized as the internal control for normalization. The expression level of 0-DPA ovule was artificially set to 1. The different color scale indicates the gene expression levels with the colors of yellow, orange, and red to present regular, moderate, and high expression abundances respectively. (**C**) MDHAR enzyme activity measurement during fiber development stages. The wild-type 0-DPA ovules and fibers at the stages of 5, 10, 15, 18, 21 and 28 DPA were collected for MDHAR enzyme activity analysis. The value of 0-DPA ovule was utilized as reference for statistical difference analysis through one-way ANOVA, with *, **, and *** to indicate the differences at 0.05, 0.01 and 0.001 levels, respectively. (**D**) AsA content and AsA/DHA ratio determinations during different fiber development stages. The materials of 0-DPA ovule and fibers at different development stages of 5, 10, 15, 18, 21 and 28 DPA were obtained for AsA content determination and AsA/DHA ratio calculation. The value of 0-DPA ovule was used as reference for statistical difference by One-way ANOVA. Asterisks indicate the significant differences, ** *p* < 0.01, *** *p* < 0.001.

The upland cotton *G. hirsutum* provides the majority of the fibers for textile industry. We analyzed the expression pattern of *MDHARs* during fiber development and did tissue-specific and fiber-developmental expression analyses in *G. hirsutum GhMDHARs*. The results indicated that *GhMDHAR1A/D* and *GhMDHAR3A/D* were preferentially expressed in all detected tissues including roots, stems, leaves, petals, stamens, pistils, ovules and fibers, with the most significant accumulations in fiber development, suggesting their potential important roles in tissue development especially in fiber growth (Figure 4A). *GhMDHAR2A/D* indicated moderate expression in all tissues, with the exception of their expressions in fibers and of *GhMDHAR2A* expression in flower tissues, implying a possible functional difference in flower development. *GhMDHAR5A/D* also showed moderate expression with an especially high enrichment in flower tissues, presenting their potential function in flower development. *GhMDHAR4A/D* and *GhMDHAR6A/D* displayed consistent expression with a moderate and low expression, respectively. Meanwhile, in the other three cotton species, *G. barbadense*, *G. arboreum* and *G. raimondii*, the expression of *MDHARs* showed highly similar levels with that of *G. hirsutum GhMDHARs* (Supplementary Figure S5), implying the diverse and important roles of *MDHAR* expressions for both fiber growth and tissue development.

To further investigate the possible role of *GhMDHARs* in fiber development, the expressions of *GhMDHAR* genes during different fiber development stages were detected by quantitative real-time polymerase chain reaction (qRT-PCR). The results indicated that, during different fiber developmental stages, *GhMDHAR3A/D* showed the most significant accumulations in 5–21 days post anthesis (DPA) fibers (the fast elongation stages), with the peak value at 15 DPA. *GhMDHAR1D* also presented moderate upregulated expression levels in 5–21 DPA fibers. *GhMDHAR1A* and *GhMDHAR4A/D* were mainly enriched in 15-DPA fibers (Figure 4B). These results suggest that these *GhMDHAR* genes might play important function for fiber development.

As MDHARs have a main function as catalytic enzymes, MDHAR activity was measured during fiber development stages. MDHAR activity indicated significant accumulation in 5–21 DPA fibers and reached a peak value at 15 DPA, compared to that in 0-DPA ovules (Figure 4C). Further, AsA content was measured to validate the key role of MDHAR in AsA regeneration. The results showed that AsA content maintained a high level in 10–21 DPA fibers (the fiber fast elongation and secondary wall deposition stages) and reached the highest level in 15-DPA fibers. The AsA/DHA ratio showed a similar tendency with significant increase at 10–21 DPA with the peak in 15-DPA fibers (Figure 4D). These results demonstrated that a higher MDHAR activity is associated with fiber growth by catalyzing AsA recycling and maintaining the AsA-mediated cellular redox status.

### 2.5. Expression Analysis of GhMDHARs under Abiotic and Phytohormone Treatments

**Figure 5:**
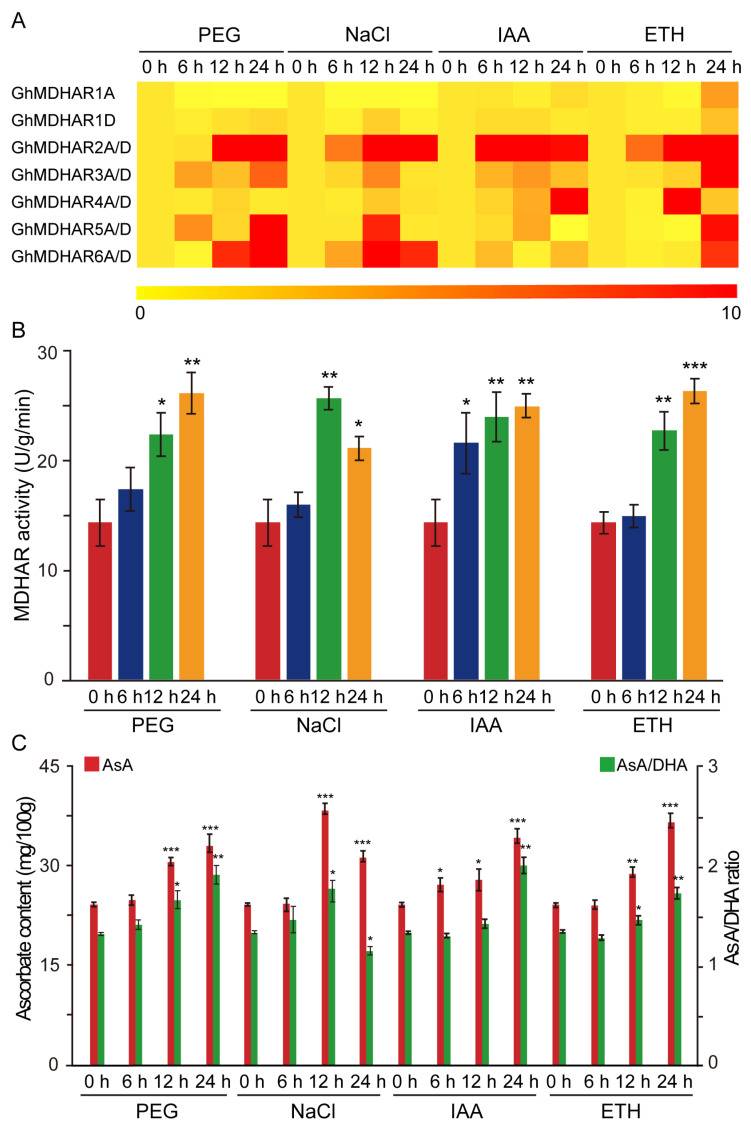
Expression analysis of *GhMDHARs* under abiotic stress and hormone treatment. The cotton leaf materials under the stresses of polyethylene glycol (PEG) and NaCl and the hormone treatments of indole-3-acetic acid (IAA) and ethylene (ETH) were collected and used for analyses of qRT-PCR, enzyme activity and AsA content, and AsA/ratio. (**A**) qRT-PCR-based heatmap of expression levels of *GhMDHARs*. The presented cotton leaf materials, with or without the treatments, were collected and extracted for RNA samples that were then used for qRT-PCR assay. The results were normalized using *GhUBQ7* gene as internal control. The expression levels of untreated 0-h materials were artificially set to 1. The different color scale represents the distinct expression abundances with the colors of yellow, orange, and red to indicate regular, moderate, and high expression respectively. The heatmap was generated by R package. (**B**) Enzyme activity determination of MDHAR. The leaves treated by the stress and hormone treatments were utilized for enzyme solution extract and then for activity measurement. (**C**) Analysis of AsA content and AsA/DHA ratio under the stress and hormone treatments. Significant difference test was performed by one-way ANOVA using the value of untreated materials (0 h) as reference, with *, **, and *** to represent the differences at 0.05, 0.01 and 0.001 levels, respectively.

Expression of *MDHAR* is regulated by environmental stress, especially drought and salt [25,52], and AsA biosynthesis and metabolism are controlled by phytohormone indole-3-acetic acid (IAA) and ethylene (ETH) [4,16,50]. Thus, to investigate the *GhMDHAR* expression features under treatments of abiotic stressors, polyethylene glycol (PEG) and NaCl and plant hormones of IAA and ETH treatments were used, and expression abundance of *GhMDHAR* genes was detected by qRT-PCR, using 0-h expression level of untreated samples as control. The *GhMDHAR* genes exhibited diverse expression patterns under different treatment conditions. *GhMDHAR2A/D*, *GhMDHAR5A/D*, and *GhMDHAR6A/D* indicated significant accumulated expression under these four treatments, at least in one treated time point, with the exceptions of *GhMDHAR5A/D* that showed moderate enrichment and of *GhMDHAR6A/D* that also showed moderate regulation under IAA treatment (Figure 5A). *GhMDHAR3A/D* displayed a medium accumulation upon PEG, NaCl, and IAA treatment, but was enriched highly under the 24-h ETH treatment. *GhMDHAR4A/D* illustrated significant increased expression under the treatments of 24-h IAA and 12-h ETH without obvious difference following PEG and NaCl stresses (Figure 5A). The MDHAR family demonstrated significant increased enzyme activity under these four conditions (Figure 5B). The contents of AsA, together with the AsA/DHA ratio, indicated a highly consistency with MDHAR activity, showing significant accumulations under conditions of PEG, NaCl, IAA, and ETH treatments (Figure 5C). These results imply that, *GhMDHARs* have functional diversity and may play an important role for cotton plants to respond to environmental stress and phytohormone treatment.

### 2.6. Analysis of GhMDHAR Involving in Fiber Elongation Development

**Figure 6:**
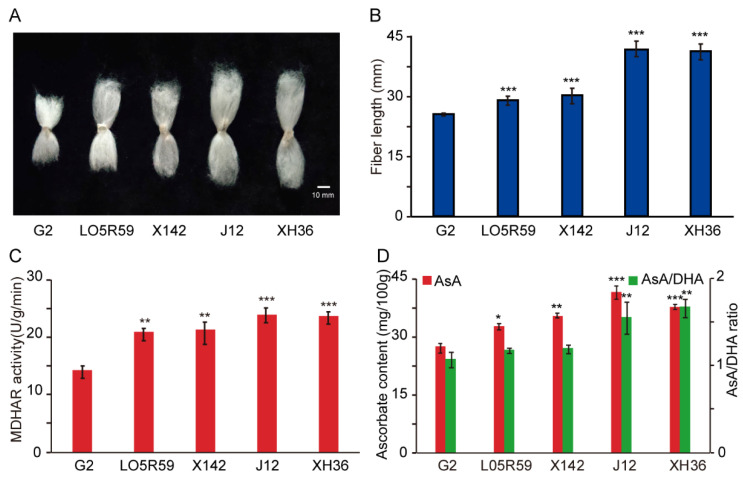
Correlation analysis between GhMDHAR expression and fiber elongation development. The fiber materials of cotton cultivars that have different fiber lengths of short-fiber G2, moderate-fiber LO5R59 and X142, and long-fiber J12 and XH36, were selected for analysis of MDHAR activity, AsA content and AsA/DHA ratio. Phenotype (**A**) and fiber length statistics (**B**) of mature fibers of different fiber-length cotton cultivars. (**C**) MDHAR enzyme activity measurement of fibers of different fiber-length cotton cultivars. The 15-DPA fibers of presented different cotton cultivars were collected and used for MDHAR activity analysis. (**D**) Determination of AsA content and AsA/DHA ratio in fibers of different fiber-length cotton cultivars. The 15-DPA fibers of different cotton cultivars were utilized for AsA content and AsA/DHA ratio analysis. The values of the short-length cultivar G2 were selected as control for statistics difference analysis by one-way ANOVA. Asterisks indicate the significant differences, * *p* < 0.05, ** *p* < 0.01, *** *p* < 0.001.

Our previous studies reported AsA biosynthesis and metabolism as positive regulators involved in fiber growth and cell elongation [16,50]. Therefore, to investigate the GhMDHAR potential function for fiber development, cotton cultivars that have short- (G2), medium- (LO5R59 and X142), and long-fiber (J12 and XH36) lengths were utilized to analyze the connection between MDHAR expression and fiber growth. As presented in Figure 6A,B, these cotton cultivars showed significant differences in fiber length. Meanwhile, MDHAR enzyme activity significantly increased and the AsA content and the AsA/DHA ratio were also significantly increased in the medium and long-fiber cultivars, compared to that in the short-fiber G2 (Figure 6C,D). These results provide a close link between high MDHAR expression and fiber development, suggesting a possible important role for MDHAR in fiber cell elongation via regulation of AsA generation and control of AsA-mediated redox status.

### 2.7. Analysis of AsA Antioxidative Enzymes under H_2_O_2_ Stimulation

**Figure 7:**
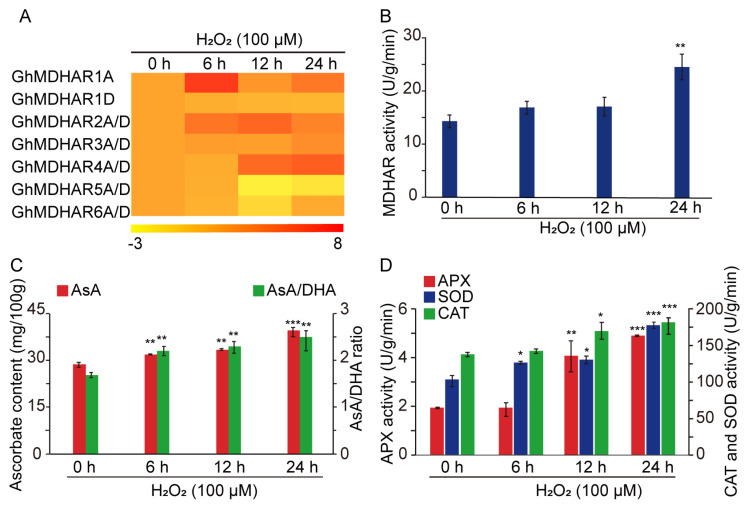
Analyses of *GhMDHAR* expression and AsA antioxidant enzyme activity under H_2_O_2_ treatment. The cotton leaves treated by 100 μM H_2_O_2_ for 6, 12 and 24 h were applied for the determinations. (**A**) Heatmap of *GhMDHARs* expression levels detected by qRT-PCR. The colors of yellow, orange, and red indicate low, moderate, and high expression levels respectively. *GhUBQ7* gene was used as internal control for normalization and the untreated 0-h expression level was artificially set to 1. R package was utilized to generate the heatmap. (**B**) MDHAR enzyme activity analysis. (**C**) Measurement of AsA content and AsA/DHA ratio. (**D**) Activity analysis of AsA antioxidant enzymes. The activities of enzymes of ascorbate peroxidase (APX), superoxide dismutase (SOD) and catalase (CAT) were measured. The values of untreated 0-h materials were used as reference for statistical difference analysis, with *, **, and *** to present the differences at 0.05, 0.01 and 0.001 levels, respectively.

With regard to the key role of ROS in plant cell and fiber cell elongation [4,16,48,53,54], and to the important function of the reductive molecule AsA and the corresponding antioxidative enzymes in redundant ROS elimination during the fiber development process [4], expressions of *GhMDHAR* and the antioxidative enzymes were measured following H_2_O_2_ treatment. The results indicated that, at the transcription level, the expressions of *GhMDHAR2A/D* and *GhMDHAR4A/D* were significantly induced after 6-h H_2_O_2_ stimulation (Figure 7A), implying a potential crucial function in response to ROS. Contrary to the expression level of untreated material, the H_2_O_2_-treated materials showed a significant promotion of MDHAR activity after 24-h supplement, together with high levels of AsA content and AsA/DHA ratio after 6-h addition of H_2_O_2_, with a continuous increased level thereafter (Figure 7B,C). Other antioxidative enzyme activities were measured. Superoxide dismutase (SOD) showed a prompt increase after 6-h H_2_O_2_ treatment and APX and catalase (CAT) indicated significant promotions after 12-h H_2_O_2_ stimulation (Figure 7D). These results demonstrate potential *GhMDHAR* candidates to participate in H_2_O_2_ response, and indicate that MDHAR, its catalyzed AsA regeneration and the successive cellular redox situation, combined with the significantly increased activity of the antioxidative enzymes, might play important role in detoxification of ROS.

## 3. Discussion

MDHAR is an essential enzyme for the regeneration of AsA that performs multiple roles in cell metabolism and physiological response as antioxidative small molecules [55,56], and thus plays important roles in plant development and cell growth [13,15,16] via a nicotinamide adenine dinucleotide phosphate (NADPH)-dependent redox reaction. A plethora of *MDHAR* genes have been identified in plants including pea, tomato, spinach, cucumber, soybean and potato [32,33,34,35,36,37]. Despite three *PpMDHAR* in *Physcomitrella patens* and six *AtMDHARs* in *A. thaliana* being reported, there are rare studies for the *MDAHR* family genes at the genome-wide level with a comprehensive systematic analysis. In this work, by a genome-wide analysis in four cotton species of *G. arboreum*, *G. raimondii*, *G. hirsutum* and *G. barbadense*, a total of 36 *MDHAR* members (Supplementary Table S1) were identified that located onto different chromosomes, with six members in diploid cotton *G. arboreum* and *G. raimondii*, and 12 members in allotetraploid cotton *G. barbadense* and *G. hirsutum*, respectively (Figure 1).

Plant MDHARs are typical FAD monomeric enzymes that catalyze redox reactions using FADH_2_ as substrate to reduce MDHA to AsA via a GSH-coupled pathway [6,9]. Cotton MDHARs have a representative FAD/NAD binding domain (Supplementary Figure S2) and conserved motifs (Figure 3C), which endow them with the function to catalyze the AsA recycling. Phylogenetic tree analysis constructed by a total of 70 MDHAR sequences from the four cotton species and eight other plant species resulted in three groups, implying a consistent function of the plant MDHARs in the same groups. The 36 cotton *MDHARs* were classified into different branches with the distinct gene structure organization and motif distribution in different branches (Figure 3), suggesting their potential diverse functions. It was established that the MDHARs that target to different cell compartments perform different functions such as cytosolic PpMDHARs for stress protection [39], peroxisomal PsMDHAR for high light intensity adaptation [57], and chloroplastic LeMDHAR for photoinhibition alleviation [23]. Cotton MDHARs showed different predicted subcellular locations (Figure 3C), maintaining a high consistency with the groups of the phylogenetic tree (Figure 2), implying, again, possible multiple functions of the different targeted MDHARs.

There is abundant evidence that *MDHAR* genes are expressed correlatively with plant development. Induced expression of pea *MDHAR* performed important function for seed germination and seedling growth through regulating AsA content [58]. *AtMDHAR4* expression is essential for seedling growth through supply of carbon skeletons and energy, via protection of the seed storage oil hydrolysis process by controlling peroxisome ROS levels [29]. In this study, *GhMDHARs* displayed diverse expression features in different cotton tissues (Figure 4A), suggesting their potential diverse functions in tissue development and plant growth. Considering that AsA is the crucial substance for plant cell enlargement and expansion [13,56], as the important AsA recycling enzyme, several GhMDHARs presented significantly promoted expressions during fiber cell development stages, with the highest level in the fast-elongating stage of 15 DPA, at both mRNA and enzyme activity levels (Figure 4A,B), suggesting there exists a close correlation between *MDHAR* expression and fiber growth.

MDHAR has been widely studied, especially in the field of plants that resist environmental stress [59]. Eight cotton genes *GhMDHAR2A/D*, *3A/D*, *5A/D* and *6A/D* indicated significant induced expressions, at both transcriptional and enzymatic levels, under the stresses of PEG and NaCl (Figure 5 A,B). *P. patens PpMDHARs* were differentially induced with diverse expression patterns under different stress conditions [39]. *Avicennia marina AmMDHAR* is a salt-inducible gene located in the chloroplast, and its overexpression in tobacco enhanced the tolerance of transgenic plants by increasing the activity of AsA-GSH antioxidative enzymes [52]. Both the mRNA and enzyme expressions of *Acanthus ebracteatus AeMDHAR* were significantly elevated in the overexpression transgenic rice plant lines that showed increased tolerance to salt stress at both germination and seedling stages [60]. The transgenic tobacco plants overexpressing arabidopsis *AtMDHAR1* exhibited strengthened resistance to salt and PEG stresses, showing an increase of MDHAR activity and reductive AsA content coupled with a higher AsA/DHA ratio and lower ROS level as the key factors for the enhanced stress tolerance [25]. In this study, under PEG and NaCl conditions, an elevation of MDHAR enzyme activity and the promotion of corresponding AsA content and AsA/DHA ratio were observed (Figure 5B,C), which displayed the positive connection between stress tolerance and AsA level increase by promotion of MDHAR expression, indicating consistency with reported studies. Phytohormones have long been established as crucial factors for plants to sense and resist the environmental stress [61]. Our results detected the rapid significant induced expressions of *GhMDHAR2A/D* and *4A/*D under IAA treatment and of *GhMDHAR2A/D*, *3A/D*, *4A/D*, *5A/D* and *6A/D* under ETH stimulation, together with the coincident synchronous increases of AsA and AsA/DHA value, validating again the close correlation between phytohormone and stress perception in plants.

AsA plays multifunction roles for redundant ROS detoxification, cell expansion or enlargement, and plant development [13,15,56]. A higher AsA content that might be catalyzed by increased MDHAR activity in longer fibers was observed (Figure 6), which indicated a similar result of AsA accumulation in elongating fibers in cotton [50,62]. Our former studies discovered that AsA accumulation, biosynthesis and metabolism play a key role for fiber cell growth [4,50]. The cell wall is the crucial factor to decide cell length and mechanical strength and is affected by wall-localized hydroxyproline rich glycoproteins (HRGPs) [7]. AsA could influence cell wall alterations as a cofactor of HRGP to promote cell elongation [63,64,65,66], suggesting the possibility of MDHAR participating in cell elongation through the involvement of cell wall assembly indirectly by catalyzing AsA regeneration. ROS, as oxidative molecules, are decisive factors for both plant cell growth and fiber elongation development by affecting redox homeostasis of the cells [4,48,53,67]. Under H_2_O_2_ treatment, the MDHAR expression, AsA content and AsA/DHA value, together with AsA-mediated antioxidative enzymes, were significantly elevated (Figure 7), indicating a close link between MDHAR and ROS signaling. Despite the known role of AsA as a reducing agent to respond to ROS, other oxidative molecules were generated by AsA-mediated oxidation reactions, suggesting another possibility for MDHAR involvement in cell elongation via redox signaling pathways [4,16].

Plant hormones such as ethylene and IAA have been reported to control plant root hair, hypocotyl development, and fiber cell elongation [68,69,70,71,72,73,74,75,76,77]. The content of IAA significantly accumulated in the fiber initiation stage and performed important functions for fiber quality [74]. An ethylene signal was the crucial element to decide fiber growth [71,72,75]. Cotton *GhMDHARs* were significantly induced by IAA and ETH (Figure 5A), coupled with the existing hormone responsive *cis*-elements (Supplementary Figure S4), showing their potential regulatory role as downstream signaling factors. As a cofactor of 1-aminocyclopropane-1-carboxylic acid oxidase (ACO), which is the key enzyme for ethylene biosynthesis, AsA may control plant cell development [78,79,80], suggesting again the correlation of MDHAR and ethylene signaling. With regard to the existing crosstalk between ROS and phytohormones, and to different subcellular distributions of MDHARs, further investigation into their synergies and functions in different cellular compartments are expected. In conclusion, our results for the first time provide comprehensive systemic analysis of the *MDHAR* gene family in plants and the four *Gossypium* species and indicate that MDHAR may be involved in fiber elongation development via controlling AsA-recycling-mediated cellular redox homeostasis.

## 4. Materials and Methods

### 4.1. Sequence Acquirement and Chromosomal Distribution of MDHAR Genes in Cotton

The cotton MDHAR sequences were obtained by submitting arabidopsis MDHARs to the genome database for *G. arboreum* (NCBI, https://www.ncbi.nlm.nih.gov/genome/) [81], *G. barbadense* (COTTONGEN, https://www.cottongen.org/) [82], *G. hirsutum* (JGI, https://genome.jgi.doe.gov/portal/) [83] and *G. raimondii* (JGI, https://genome.jgi.doe.gov/portal/) [84] using local BLASTP program. The transcriptome data of *G. hirsutum* were downloaded from NCBI (https://www.ncbi.nlm.nih.gov) SRA database (PRJNA248163). The RNA-Seq clean data were mapped to the presented *Gossypium* genome by TopHat2 (Version 2.0.13, Center for Bioinformatics and Computational Biology, Maryland, USA) and the fragments per kilobase of exon per million reads mapped (FPKM) value was calculated by Cufflinks (Version 2.1.1, University of California, California, USA) [85,86]. The MDHAR candidates were then confirmed by screening the conserved domains by online InterProScan at the website http://www.ebi.ac.uk [87]. The theoretical molecular weight (MW) and isoelectric point (*p*I) were calculated by online tools at ExPASy website (https://www.expasy.org). Subcellular localizations of the MDHAR proteins were predicted by the online tool ProtComp at Softberry server (http://www.softberry.com/). The MDHAR chromosomal distributions were described by Mapinspect software (Version1.0, Wageningen university, Ralph van Berloo, the Netherlands).

### 4.2. Evolutionary Analysis and Promoter Cis-Element Identification

Multiple sequence alignment of the MDHAR proteins was performed using ClustalW and the phylogenetic tree of the MDHAR proteins from four cotton cultivars and another eight plant species was constructed by Molecular Evolutionary Genetics Analysis (MEGA 7.0, Institute for Genomics and Evolutionary Medicine, Temple University, Philadelphia, USA) with the parameters of neighbor-joining (N—J) method of 1000 bootstrap replicates, model of p-distance, and Gaps/Missing data treatment by Pairwise deletion. The exon-intron distribution and conserved motif analyses were recognized using the online software of the gene structure display server (GSDS, http://gsds.cbi.pku.edu.cn/index.php) and Multiple EM for Motif Elicitation (MEME, http://meme-suite.org/tools/meme) respectively [88,89]. The *Ka*/*Ks* was calculated by Dnasp 5.0 software (Version 5.0, Universitat de Barcelona, Barcelona, Spain) [90]. The *Ka*/*Ks* ratios for the *GhMDHAR* genes were calculated to assess the selection pressure on duplicated genes, with *Ka*/*Ks* ratio >1, <1, or = 1 to indicate positive selection, negative selection and purify evolution, respectively [91]. The Tajima relative rate test were used to estimate the evolutionary rate by MEGA 7.0 software. The Circos software (Canada’s Michael Smith Genome Sciences Center, Vancouver, BC, Canada) was used for collinear analysis and the collinear file was calculated by Multiple Collinearity Scan toolkit (MC Scan, Plant Genome Mapping Laboratory, University of Georgia, Georgia, USA) [92]. The TBtools software (State Key Laboratory for Conservation and Utilization of Subtropical Agro-Bioresources, Guangdong, China) was utilized to extract the 1500 bp promoter region upstream of start codon which was then submitted to the online software PlantCARE (http://bioinformatics.psb.ugent.be/webtools/plantcare/) for analyzing the *cis*-acting element composition [93].

### 4.3. Cotton Planting and Material Treatment

Upland cotton (*G. hirsutum*) varieties (X142, G2, LO5R59, J12) and Sea-island cotton (XH36) plants were cultivated in the experimental field of Shihezi University in Shihezi city, Xinjiang, China. The materials of X142 were collected for gene expression analysis, enzyme activity measurement, and treatments of stress and phytohormone. Cotton bolls of X142 were marked with a label on the day post anthesis (defined as 0 DPA) and the ovules and fibers were then collected in different developmental time points of 5, 10, 15, 18, 21 and 28 DPA for cryopreservation and successive use. Cotton seeds were sown in nutrient soil and planted in the greenhouse at 28 °C with a photoperiod of 16 h light/8 h dark. The leaves of three-week-old seedlings of X142 were treated with 20% polyethylene glycol (PEG), 100 mM sodium chloride (NaCl), 100 µm indole-3-acetic acid (IAA), 100 µm ethephon (ETH), and 100 mM H_2_O_2_ for 0, 6, 12, 24 h respectively. The treated leaf materials of X142 were collected and stored at −80 °C after flash-freezing in liquid nitrogen for further utilization. The matured fibers of different cotton varieties (G2, LO5R59, X142, J12, and XH36) were collected for length measurement by three independent experiments with 30 samples for each replicate.

### 4.4. RNA Extraction and qRT-PCR Analysis

Total RNA was extracted from different cotton tissues by our previous method as described [4,16]. The cDNA synthesis was obtained through reverse transcription reaction with 5 microgram total RNA as material according to the instruction of SuperScript^®^ III (Invitrogen, Carlsbad, CA, USA), and then used as a template for qRT-PCR detections with SYBR green real-time PCR master mixes (Applied biosystems, Foster, CA, USA). The specific primers were provided in Supplementary Table S5 and the relative expression levels of the target genes were calculated through the 2^−ΔΔCt^ method using the *GhUBQ7* gene as internal control for normalization [94,95]. The relative expression levels of *GhMDHAR* genes measured by qRT-PCR were used to generate a heatmap that visualized by MultiExperiment viewer (MeV, version 4.9, J. Craig Venter Institute, La Jolla, CA, USA) software.

### 4.5. Enzyme Assays

The MDHAR enzyme activity was measured by a spectrophotometric method. The materials were ground to powder in liquid nitrogen, and about 50 mg of raw powder was added into 1 mL of pH 6.0 50 mM potassium phosphate buffer (including 1 mM AsA, 40 mM KCl, and 2 mM CaCl_2_) that was then centrifuged at 14,000 rpm at 4 °C for 10 min. The reaction mix was obtained by the addition of collected enzyme extracts in pH 7.6 50 mM potassium phosphate buffer (containing 0.25 mM NADH, 2.5 mM AsA, and 15 μM FAD), and enough ascorbate oxidase (0.5 unit) was added into the mix solution to trigger the reaction. The values were detected at 340 nm for 2 min, and the absorbance decrease generated by the oxidation of NADH was used to calculate the MDHAR enzyme activity with the extinction coefficient of 6.22 mM^−1^·cm^−1^ [96]. The other regular antioxidant enzyme activities of ascorbate peroxidase (APX), superoxide dismutase (SOD), and catalase (CAT) were measured by commercial kits provided by the biotechnological company (Solarbio, Beijing, China).

### 4.6. Measurement of Asc, AsA and DHA

Asc measurement was performed as in our previous method [16]. Briefly, 5 g of material was ground in mortar on ice in 20 mL 50 g/L trichloroacetic acid (TCA) solution with successive dilution to 100 mL 50 g/L TCA solution. The collected supernatant by filtration was used for AsA determination. The reaction components included 1 mL 50 g/L TCA solution, 1 mL sample extract, 1 mL absolute ethanol, 0.5 mL 0.4% phosphate-ethanol, 1 mL 5 g/L BP-ethanol, and 0.5 mL FeCl_3_-ethanol solution. The total AsA content was generated by detecting the values at 534 nm through spectrophotometry according to the Asc content standard curve. The 0.5 mL of 60 mmol/L dithiothreitol (DTT) was added to the reaction mix for total Asc determination.

## Data Availability

No new data were created or analyzed in this study. Data sharing is not applicable to this article.

## Supplementary Materials

The following are available online at https://www.mdpi.com/2223-7747/10/2/227/s1. Supplementary Figure S1: Segmental duplication of *MDHARs* and syntenic analysis of *G. arboreum*, *G. raimondii*, *G. hirsutum* and *G. barbadense*. Supplementary Figure S2: Amino acid sequence alignment of the 36 MDHARs in the four cotton species *G. arboreum, G. raimondii, G. hirsutum* and *G. barbadense*. Supplementary Figure S3: Detailed amino acid composition of the nine conserved motifs distributed in *MDHAR* proteins. Supplementary Figure S4: Analysis of *cis*-acting element distributions in the promoter regions of *MDHAR* genes in four cotton species of *G. arboreum*, *G. raimondii*, *G. hirsutum* and *G. barbadense*. Supplementary Figure S5: Heatmap of expression levels of *G. barbadense GbMDHARs* in different tissues. Supplementary Table S1: The *MDHAR* genes information and properties of the deduced proteins. Supplementary Table S2: Tajima relative rate tests of *GhMDHAR* duplicated gene pairs. Supplementary Table S3: The *Ka*/*Ks* ratios for duplicate *MDHAR* genes in cotton. Supplementary Table S4: Putative *cis*-element sequences in promoter regions of *MDHAR* genes. Supplementary Table S5: Primers used in this work.

## Abbreviations

ACO	1-aminocyclopropane-1-carboxylic acid oxidase
ANOVA	Analysis of Variance
AO	Ascorbate oxidase
APX	Ascorbate peroxidase
AsA	Ascorbic acid
Asc	Ascorbate
CAT	Catalase
DHA	Dehydroascorbic acid
DPA	Day post anthesis
DTT	Dithiothreitol
ETH	Ethephon
FAD	Flavin adenine dinucleotide
FPKM	Fragments per kilobase of exon per million reads mapped
GSDS	Gene Structure Display Server
GSH	Glutathione
HRGP	Hydroxyproline rich glycoprotein
IAA	Indole-3-acetic acid
Ka	Nonsynonymous substitution rate
kDa	KiloDalton
Ks	Synonymous substitution rate
MDHAR	Monodehydroasorbate reductase
MEGA	Molecular evolutionary genetics analysis
MEME	Multiple EM for Motif Elicitation
MIPS	Myo-inositol-1-phosphate synthase
MW	Molecular weight
NAD	Nicotinamide adenine dinucleotide
NADH	Nicotinamide adenine dinucleotide
NADPH	Dinucleotide phosphate
NCBI	National Center for Biotechnology Information
N—J	Neighbor-joining
ORF	Open reading frame
PEG	Polyethylene glycol
*p*I	Isoelectric point
qRT-PCR	Quantitative real-time polymerase chain reaction
ROS	Reactive oxygen species
SOD	Superoxide dismutase
TCA	Trichloroacetic acid
TNT	2,4,6-trinitrotoluene
UBQ	Ubiquitin
