# Supplementary material for: Genome-Wide Analysis of MDHAR Gene Family in Four Cotton Species Provides Insights into Fiber Development via Regulating AsA Redox Homeostasis
Source: Plants (Basel). 2021 Jan 25;10(2):227. doi: 10.3390/plants10020227 (PMC7912408; doi:10.3390/plants10020227)
Supplement: Supplementary file 1 [file plants-10-00227-s001.zip › Supplementary materials_20210117/Supplementary figures and tables_20210117/Supplementary figures and legends_revision2_20210117.doc]

**Supplementary Figures and legends**

**Supplementary Figure S1.** Segmental duplication of *MDHARs* and syntenic analysis of *G. arboreum*, *G. raimondii*, *G. hirsutum*, and *G. barbadense*. All the chromosomes from the four cotton species *G. arboreum*, *G. raimondii*, *G. hirsutum*, and *G. barbadense* are showed in yellow, green, blue, and red circular columns, respectively. The *MDHAR* positions are indicated by short black lines. The duplicated *MDHARs* are linked by purple lines in *G. arboreum* and *G. raimondii*, and by yellow lines in *G. hirsutum* and *G. barbadense*,respectively. Syntenic relationships are indicated by diverse colored lines presented between each two cotton species.

**Supplementary Figure S2.** Amino acid sequence alignment of the 36 MDHARs in the four cotton species *G. arboreum*, *G. raimondii*, *G. hirsutum*, and *G. barbadense*. Each MDHAR contains the FAN/NAD-binding domian that was indicated by black line.

**Supplementary Figure S3.** Detailed amino acid composition of the nine conserved motifs distributed in MDHAR proteins.

**Supplementary Figure S4.** Analysis of *cis*-acting element distributions in the promoter regions of *MDHAR* genes in four cotton species of *G. arboreum*, *G. raimondii*, *G. hirsutum*, and *G. barbadense*. The specific *cis*-acting element distributions in the promoters of *MDHARs* of *G. arboreum*, *G. raimondii*, *G. hirsutum*, and *G. barbadense* were illustrated, showing the consistent classification into three groups with that in Figure 2. The promoter sequences were indicated by gray lines and the distinct elements were showed by different colored short vertical lines with the details listed in Supplementary Table S4.

**Supplementary Figure S5.** Heatmap of *MDHAR* expression levels of *Gossypium barbadense*, *G. arboreum*, and *G. raimondii* in different tissues. The FPKM values in different tissues including root, stem, leaf, petal, stamen, pistil, ovule, and different-stage fiber and seed were extracted from the public transcriptomic data released at NCBI website and then were used to generate the heatmap by R language. The blue, yellow, and red colors indicate the low, moderate and high expression levels respectively.
