# Supplementary material for: Genome-Wide Analysis of MDHAR Gene Family in Four Cotton Species Provides Insights into Fiber Development via Regulating AsA Redox Homeostasis
Source: Plants (Basel). 2021 Jan 25;10(2):227. doi: 10.3390/plants10020227 (PMC7912408; doi:10.3390/plants10020227)
Supplement: Supplementary file 1 [file plants-10-00227-s001.zip › Supplementary materials_20210117/Supporting figure and table_20210106/Supporting Figure_correlation analysis_20210106.doc]

**Supporting Figure S1.** Expression **c**orrelation analysis between *GhMDHARs* and the genes of ascorbate metabolism. The genes of *Gossypium hirsutum* involved in ascorbate (Asc) biosynthesis (*vitamin C*, *VTC*), Asc oxidation (*ascorbate oxidase*, *AO*), and Asc recycling (*ascorbate peroxidase*, *APX*; *superoxide dismutase*, *SOD*; and *catalase*, *CAT*), were selected for correlation analysis with *GhMDHARs*. The FPKM value of all *GhMDHARs* and the ascorbate metabolism related genes that were detectable in the transcriptome data was analyzed by SPSS 16.0 for calculating the correlation efficiency. Red and blue denote the high and low correlation efficiency respectively.
